# Arginine methyltransferase CARM1/PRMT4 regulates endochondral ossification

**DOI:** 10.1186/1471-213X-9-47

**Published:** 2009-09-02

**Authors:** Tatsuo Ito, Neelu Yadav, Jaeho Lee, Takayuki Furumatsu, Satoshi Yamashita, Kenji Yoshida, Noboru Taniguchi, Megumi Hashimoto, Megumi Tsuchiya, Toshifumi Ozaki, Martin Lotz, Mark T Bedford, Hiroshi Asahara

**Affiliations:** 1 Department of Molecular and Experimental Medicine, The Scripps Research Institute, La Jolla, CA 92037, USA; 2 Department of Carcinogenesis, University of Texas M. D. Anderson Cancer Center, PO Box 389, Smithville, TX 78957, USA; 3 Department of Orthopedic Surgery, Graduate School of Medicine, Dentistry and Pharmaceutical Sciences, Okayama University, Okayama 700-8558, Japan; 4 Department of Regenerative Medicine, National Research Institute for Child Health and Development, Tokyo 157-8535, Japan

## Abstract

**Background:**

Chondrogenesis and subsequent endochondral ossification are processes tightly regulated by the transcription factor Sox9 (SRY-related high mobility group-Box gene 9), but molecular mechanisms underlying this activity remain unclear. Here we report that coactivator-associated arginine methyltransferase 1 (CARM1) regulates chondrocyte proliferation via arginine methylation of Sox9.

**Results:**

CARM1-null mice display delayed endochondral ossification and decreased chondrocyte proliferation. Conversely, cartilage development of CARM1 transgenic mice was accelerated. CARM1 specifically methylates Sox9 at its HMG domain *in vivo *and *in vitro*. Arg-methylation of Sox9 by CARM1 disrupts interaction of Sox9 with beta-catenin, regulating *Cyclin D1 *expression and cell cycle progression of chondrocytes.

**Conclusion:**

These results establish a role for CARM1 as an important regulator of chondrocyte proliferation during embryogenesis.

## Background

The precise patterning of the developing skeletal framework relies on the appropriate control of chondrogenesis, a multistep process during which mesenchymal cells differentiate into chondrocytes[1,2]. This process is tightly regulated by transcription factors, including Sox9 [3-6]. Mice lacking Sox9 display distortion of numerous cartilage-derived skeletal structures[7]. In addition, mice overexpressing Sox9 in chondrocytes show dwarfism with decreased chondrocyte proliferation and delayed endochondral bone formation[1]. However, the precise mechanism how Sox9 regulates chondrogenesis both spatially and temporally is still largely unknown.

CARM1 belongs to a family of arginine-specific protein methyltransferases (PRMTs), which includes at least eight members (PRMT1-8) [8]. All PRMT family members share a core arginine methyltransferase region composed of a conserved Ado-Met binding domain and a more divergent C-terminal domain. CARM1 has been shown to synergistically activate transcription with nuclear receptors in combination with other coactivators, such as p160 family, p300/CBP and SRC-2/TIF2/GRIP1[9,10]. After recruitment to promoters of steroid-responsive genes, CARM1 methylates specific residues (Arg17 and Arg26) at the N-terminus of histone H3 resulting in transcriptional activation[11,12].

## Results and Discussion

### Skeletal phenotype of CARM1 null mice

**Figure 1:**
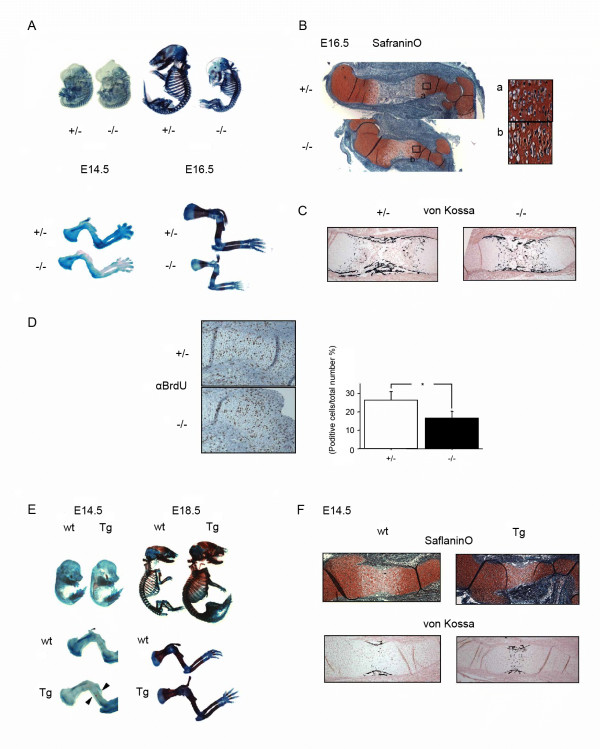
**Analysis of skeletal phenotypes in CARM1-null mutant embryos**. (A) Appearance of skeletons of E14.5 and E16.5 embryos stained with alcian blue (cartilage) followed by alizarin red (bone). Forelimbs from E16.5 wild type (wt) (top) and null (lower) siblings show shortened bones. (B) Histological analysis of limbs of CARM1-null mutant embryos. SafraninO staining of humerus of E16.5 embryos. (B: a & b) Boxed regions are shown at higher magnification. (C) Staining by von Kossa's method in the humerus of E16.5 heterozygous and mutant embryos. (D) BrdU incorporation of humerus of E16.5 embryos. Differences, assessed by one-way analysis of variance and an unpaired Student's *t*-test (*), are significant p < 0.001. (E) Whole skeletal preparations of E14.5 and E18.5 CARM1 transgenic (Tg) and wt littermates. Arrow indicates a calcificated region in the shaft of the humerus of E14.5 Tg embryos. (F) Histological analysis of limbs in E14.5 wt and Tg embryos. SafraninO staining of the humerus in E14.5 embryos. Staining using von Kossa's method visualizes mineral deposition in the humerus of E14.5 Tg embryos.

To examine the potential role of CARM1 in skeletal development, we analyzed CARM1 null embryos, which die immediately after birth as reported[13]. At E14.5, before ossification starts, embryos did not exhibit significant differences by alcian blue and alizarin red staining. However, at E16.5, ossification in null mutants was remarkably delayed, and the size of null mutant was smaller compared with heterozygotes (Figure 1A, B: SafraninO). At E18.5, the difference between null and wild type gets relatively weak (data not shown). Using von Kossa staining, we observed that absorption of calcified cells in null mutants at E16.5 was delayed (Figure 1C: von Kossa). *In situ *hybridization (ISH) of, *Col2a1*, *Col10a1*, *Bone Sialoprotein (BSP)*, *Osteopontin (Op)*, *Osteocalcin (Oc)* and *Runx2* in mutant embryos supported the conclusion that endochondral bone formation was significantly delayed (Additional file 1). Importantly, BrdU pulse-labeling of cells in E16.5 CARM1-null and wild type mouse embryos revealed a marked reduction in the number of BrdU-positive chondrocytes in mutant compared with wild type embryos (Figure 1D: áBrdU), indicating that chondrocyte proliferation in mutant embryos was inhibited.

### Bone development of CARM1-transgenic mice

For gain-of-function analysis, we generated transgenic mice in which CARM1 expression is driven by the ubiquitously expressed beta-actin promoter (Additional file 2). In contrast to null mutants, CARM1-transgenic mice analyzed at E18.5 were larger than controls (Figure 1E: Double stain, Tg). Alizarin red positive regions appeared at the shaft of humerus in E14.5 CARM1-transgenic mice but were not visible in wild type mice (Figure 1E: Double stain, Tg limb, arrow heads). von Kossa staining showed absorption of calcified regions was also accelerated in E14.5 transgenic compared with wild type mice (Figure 1F: von Kossa), while SafraninO staining was unchanged and chondrocyte differentiation marker, *Col2a1 *and *Col10a1 *expression was not significantly altered in cartilage in E14.5 transgenic compared to wild type mice (Figure 1F: SafraninO, Tg, Additional file 1). These data indicate that endochondral bone formation in CARM1 transgenic mice is accelerated relative to wild type mice, although we could not exclude the possibility that CARM1 may also directly regulate osteoblasts differentiation.

### CARM1 expression in growth plates

**Figure 2:**
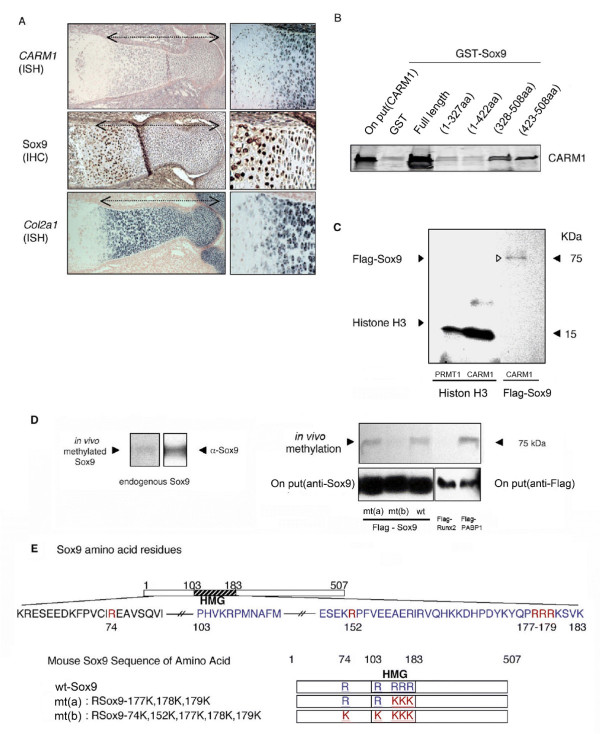
**CARM1 interacts with and methylates Sox9**. (A) Histological analysis of E15.5 wt embryos. Sections of humerus were stained with CARM1 and Col2a1 mRNA probes. Sox9 is stained by ISH and immunohistochemistry (IHC). (B) Sox9 interacts with CARM1 *in vitro*. Recombinant CARM1 protein and GST-Sox9 fragments were mixed and subjected to a GST-pull down assay. (C) Sox9 recombinant proteins and Histone H3 were incubated with PRMT1 and CARM1 in the presence of [^3^H] AdoMet. (D) Endogenous Sox9 methylation was detected in mouse primary cultured chondrocytes (left panel). Sox9 multiple point mutants a (mt(a)) and b (mt(b)), wt Sox9, Flag-tagged PABP1 (as a positive control) and Flag-tagged Runx2 (as a negative control) expression plasmids were transfected into 293T cells (right panels). (E) Schematic representation of Sox9 mutants used in the methylation assay.

To evaluate a potential functional link between Sox9 and CARM1, we examined their expression patterns during bone formation. *CARM1 mRNA* expression was high in proliferating chondrocytes of growth plates. Immunohistochemistry of Sox9 and ISH analysis of *Col2a1* show that Sox9 and *Col2a1* expression overlaps with that of *CARM1* at the proliferating zone of wild type E15.5 growth plates (Figure 2A), although chondrocytes at the prehypertrophic zone express lower levels of CARM1 but relatively abundant Sox9 and Col2a1[14].

### Sox9 and CARM1 interaction

We next asked whether CARM1 and Sox9 interact. In a GST-pull down assay, full length GST-Sox9 protein bound to *in vitro *translated CARM1 protein, suggesting direct interaction (Figure 2B: GST-pull down). To define the interaction domain of Sox9 with CARM1, we performed the assay using *in vitro *translated CARM1 and bacterially expressed GST-Sox9 fragments. Full length GST-Sox9 (1-507aa) and 328-508aa and 423-508aa fragments interacted with CARM1, while 1-327aa and 1-422aa fragments did not (Figure 2B GST-pull down). Taken together, CARM1 likely interacts with Sox9 via the Sox9 carboxy-terminal domain, which was previously reported to be a transcription activation domain[15].

### CARM1 methylates Sox9

Since CARM1 methylates some non-histone proteins, such as CBP/p300[16] and splicing factors[17], we asked whether Sox9 can serve as a substrate for CARM1. Recombinant Flag-Sox9 was incubated with recombinant CARM1/PRMT4 or PRMT1 in the presence of ^3^H-labeled S-adenosyl-L-methionine ([^3^H]AdoMet) as a methyl donor. Histone H3 was assayed as a positive control because H3 can be methylated by CARM1 and PRMT1[18]. We observed that Sox9 could be methylated by CARM1/PRMT4, but not by PRMT1 (Figure 2C). Arginine methylation was also seen in endogenous Sox9 immunoprecipitated from mouse (Figure 2D, left) or human (data not shown) primary cultured chondrocytes. A recent report showing that the HMG domain of HMGA1a can be arginine-methylated[19] prompted us to determine whether arginine residues in the HMG domain of Sox9 (74-179aa) are CARM1 targets. Based on in vitro methylation assays, simultaneous mutation of five arginine residues within the HMG domain (mt(b)) completely abolished methylation signals, whereas mutants exhibiting R177K, R178K and R179K (mt(a)) were methylated similarly to wild type (Figure 2D, right &2E). These data indicate that CARM1 methylates multiple arginine residues within the HMG domain of Sox9.

### CARM1 regulates chondrocytes proliferation

**Figure 3:**
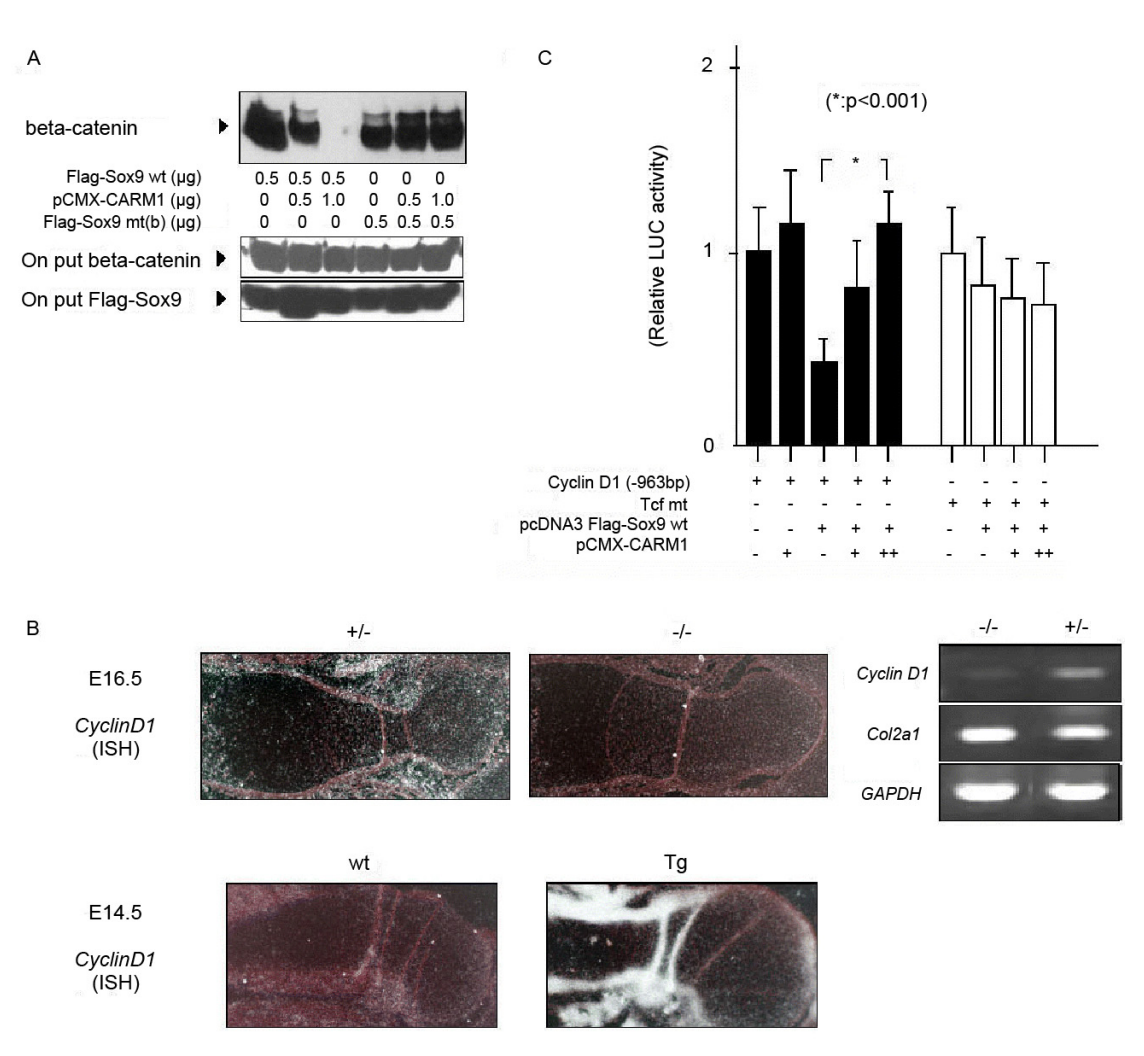
**CARM1 regulates Cyclin D1 gene expression**. (A) Lysates of SW1353 cells transfected with wt or mutant Flag-Sox9 were immunoprecipitated with anti-Flag antibody. Precipitates were subjected to Western blotting with an anti-beta-catenin (middle panel) and anti-Flag antibody (lower panel), respectively. Interaction of beta-catenin with wt Sox9 was almost abolished in the presense of high-dose CARM1 (upper panel). (B) Left: Expression of *Cyclin D1 *mRNA (ISH) in the humerus of E16.5 heterozygous, mutant and E14.5 wt and Tg embryos. Right: Expression of *Cyclin D1 *mRNA from E16.5 limb buds by RT-PCR. (C) Transcriptional regulation of the *Cyclin D1 *promoter by Flag-Sox9 and pCMX-CARM1 expression plasmids. Activating of Tcf mt reporter was also measured by a luciferase assay. Statistical significance is assessed by one-way analysis of variance and unpaired Student's *t*-test (*).

Decreased chondrocyte proliferation phenotypes seen in Sox9 transgenic mice are partly explained by the model that Sox9 competes with Tcf/Lef for binding to beta-catenin and regulates chondocyte proliferation via *Cyclin D1 *expression. The beta-catenin/Tcf complex binds the Tcf/Lef consensus site in the *Cyclin D1 *promoter, transactivating *Cyclin D1*[20]. Sox9 reportedly inhibits transcriptional beta-catenin activity, an inhibition that does not result from competition by Sox9 with Tcf/Lef for Tcf/Lef DNA-binding sites[21]. To determine whether arginine methylation of Sox9 modulates its interaction with beta-catenin, we transfected wild type and methylation point mutant forms of Flag-tagged Sox9 expression plasmids into SW1353 cells, a chondrosarcoma cell line. Immunoprecipitation analysis showed that CARM1 overexpression decreased the interaction between Flag-tagged Sox9 and endogenous beta-catenin proteins (Figure 3A: top). By contrast, protein with mutation of all Sox9 methylation sites (residues 74, 152, 177, 178, and 179, R to K) strongly interacted with beta-catenin, even when CARM1 was overexpressed, suggesting that methlyation of CARM1 inhibits the interaction between Sox9 and beta-catenin or that CARM1 binding to Sox9 blocks beta-catenin binding. We also checked whether these mutations on Sox9 may effect on cellular localization. Mutants Sox9 R177K, R178K and R179K were mainly located in the nucleus as well as wild type Sox9, when they were orverexpressed in the SW1353 cell line (data not shown).

### CARM1 regulates *Cyclin D1 *expression

*Cyclin D1 *expressed in proliferating chondrocytes of growth plates is required for normal chondrocyte proliferation[22,23]. In growth plates of E16.5 CARM1-null mutants, *Cyclin D1 *mRNA levels were reduced (Figure 3B: ISH of *CyclinD1 *in CARM1-null), whereas *Col2a1 *was expressed in the limb bud of CARM1-null mutants (Figure 3B: RT-PCR). By contrast, *Cyclin D1 *mRNA levels were remarkably increased in E14.5 CARM1-transgenic mice (Figure 3B: ISH of *Cyclin D1 *in CARM1-Tg). These data suggest that CARM1 promote *Cyclin D1 *gene expression. To determine how CARM1 regulates *Cyclin D1 *expression, we performed *Cyclin D1 *promoter assays in human embryonic kidney 293 cells, which express endogenous Lef1. On the -963CD1 *Cyclin D1 *promoter reporter plasmid and on a Tcf mt reporter plasmid with a mutation in the Tcf binding site[20], Sox9 inhibited basal promoter activity, as previously reported (Figure 3C). CARM1 overexpression rescued inhibition by Sox9 in this assay, suggesting that CARM1 may inhibit Sox9 and beta-catenin complex formation and thus increase *Cyclin D1 *expression. In contrast CARM1 and Sox9 did not activate transcription using the Tcf mt reporter plasmid, which lacks beta-catenin reactive sites. With the mutation at all 74,152,177,178,179 shows strong suppression at *CyclinD1* promoter, however, this suppression did not relieved by overexpression of CARM1 (data not shown). This suggests that CARM1 and Sox9 may cooperatively regulate *CyclinD1 *promoter activity.

**Figure 4:**
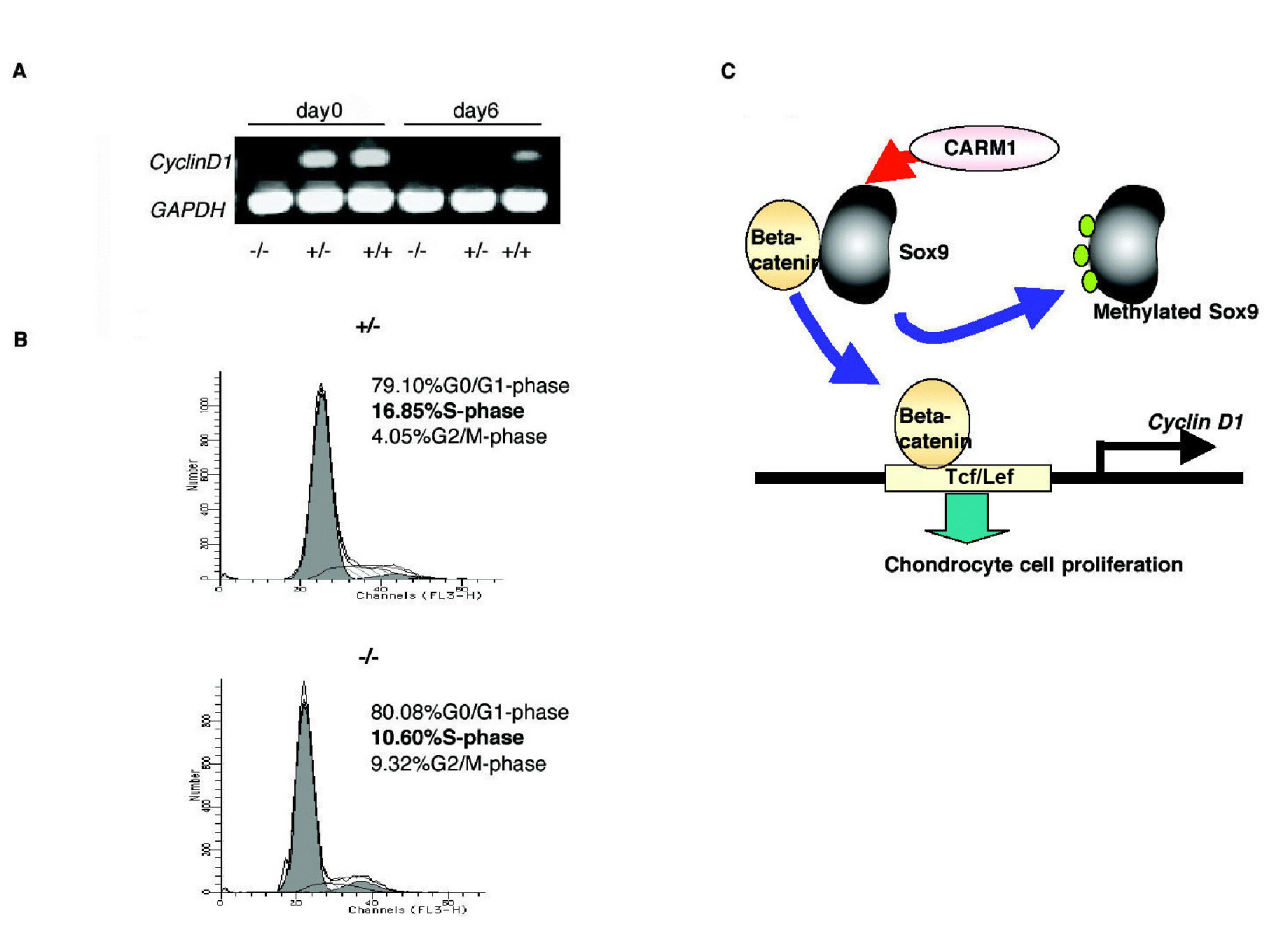
**CARM1 regulates chondrocytes proliferation**. (A) Profiles of primary cultured chondrocytes from Tg mice were evaluated at days 0 and 6. Expression change of *Cyclin D1 *mRNA during growth of chondrocytes was detected by using RT-PCR. (B) Mouse chondrocyte primary cell cultures were used for for FACS analysis. Cell cycle distribution was determined by propidium iodine staining of nuclear DNA. (C) Model showing functional and physical interactions between Sox9 and beta-catenin regulated by Sox9 methylation in chondrocytes. Interaction between Sox9 and beta-catenin is inhibited by Sox9 methylation caused by CARM1. Inhibition results in chondrocyte proliferation via up-regulation of beta-catenin/Tcf-Lef activity and *Cyclin D1 *mRNA expression.

Evidence of CARM1-dependent *Cyclin D1 *regulation prompted us to ask whether reduced chondrocyte proliferation may partly explain bone development phenotypes seen in CARM1-null mutant and transgenic embryos. Consistent with BrdU experiments (Figure 1D: BrdU stain), CARM1-null chondrocytes showed remarkably lower *Cyclin D1 *mRNA than that seen in CARM1-heterozygotes and wild type chondrocytes (Figure 4A: RT-PCR). CARM1-heterozygotes chondrocytes showed quicker disappearance of *Cyclin D1 *mRNA than wild type. Furthermore, 10.60% of CARM1-null chondrocytes were in S-phase, compared with 16.85% in heterozygous chondrocytes, a typical shift seen following *Cyclin D1 *downregulation (Figure 4B: FACS) [22]. Taken together, these data support the idea that reduced chondrocyte proliferation in CARM1-null mutant embryos is partly due to inhibition of *Cyclin D1 *expression (Figure 4C).

## Conclusion

Sox9 function is likely regulated by post-translational modifications such as phosphorylation, ubiquitination or sumoylation [24-26]. Here we demonstrate that Sox9 is Arg-methylated by CARM1. CARM1 is involved in the epigenetic programming of early embryo development[27], and also early T cell development[28]. Our study shows that CARM1 also plays a key role in cartilage development. Further analysis of the role of CARM1 on chromatinized and non-chromatinized substrates during different developmental stages should indicate the physiological role of arginine methylation signals.

## Methods

### Tissue Culture Methods and Transfections

The human chondrosarcoma cell line (SW1353) was grown in Dulbecco's modified Eagle's medium (Cellgro, Mediatech) supplemented with 10% fetal calf serum and penicillin/streptomycin (Sigma). Cells were transfected using GeneJammer (Stratagene) according to the manufacturer. Amounts of transfected plasmids were as follows: for Sox9-dependent activation, 50 ng of the *Cyclin D1 *promoter plasmids, -963CD1, Tcf mt, and 100 ng of pCDNA3-beta-catenin, 100 ng of Flag-tagged Sox9 plasmids and pCMX-CARM1 expression plasmid. Luciferase activity was normalized using 1 ng of pcDNA-LacZ as an internal control for transfection efficiency.

### Immunoprecipitations

Cells were washed once in ice-cold phosphate-buffered saline before scraping them at 4°C in 1 ml of phosphate-buffered saline. Cells were resuspended in radioimmune precipitation assay buffer (RIPA) buffer (50 mM Tris-HCl, pH 7.5, 200 mM NaCl, 1% Nonidet P-40, 1 mM EDTA, 2.5 mM EGTA, 10% glycerol, and the phosphatase inhibitors P-5726 and P-2850 (Sigma)). Cell extracts were sonicated and centrifuged at 14,000 rpm for 10 min. Supernatants were used as crude extracts for immunoprecipitations. Nonspecific binding was reduced by preincubation of extracts with protein G-Sepharose (P-4691; Sigma) for 30 min. Pellets were discarded, and extracts incubated with immune sera or controls for 2-4 h. Immunoprecipitations were performed with 5 μl of anti-Flag anti-antibody body (M2; Sigma).

### Glutathione S-Transferase (GST) Pull-downs

GST-Sox9 fusion proteins were produced in *Escherichia coli *and purified. Binding of proteins to glutathione-Sepharose was done in 20 mM Hepes, pH 7.4, 50 mM NaCl, 1 mM MgCl_2_, 0.2 mM dithiothreitol, 0.5 mM phenylmethylsulfonyl fluoride, 20 μg/ml leupeptin, 20 μg/ml aprotinin, and 0.05% Tween 20. Bound proteins were resolved on SDS-PAGE and visualized by autoradiography.

### Quantitative PCR

Poly(A)+ and total RNA were extracted from homogenized mouse embryo limbs and mouse chondrocyte cells using the Fast Track 2.0 (Invitrogen) or the RNeasy (Qiagen) kit. RNA samples were treated with DNase I (Promega), and RNA quality was assessed by gel electrophoresis. cDNA was prepared by reverse transcription of 500 ng of total RNA using the Superscript II enzyme and oligo (dT) primer (Invitrogen). Resulting cDNAs were amplified using the QuantiTect SYBR Green PCR kit (Qiagen) and the iCycler iQ Real Time PCR detection system (Bio-Rad). All mRNA expression data from quantitative PCR with reverse transcription was normalized to glyceraldehyde-3-phosphate dehydrogenase (*GAPDH*) expression in the corresponding sample.

### Histological Analysis

Whole-mount alcian blue staining of mouse embryos and alcian blue and alizarin red staining of skeletons were performed as described[24]. For histological analysis, embryos were fixed with 4% paraformaldehyde and embedded in paraffin. Sections of 4 μm were stained with SafraninO, or with the von Kossa reaction and nuclear fast red. Immunohistochemical staining was performed using peroxidase chromogens (Zymed Laboratories) and TrueBlue substrate (KPL). The following antibodies were used: goat polyclonal anti-Sox9 (1:100, Santa Cruz); rabbit polyclonal anti-Sox9 (1:100); rabbit polyclonal anti-Cyclin D1 (1:100, Santa Cruz); and mouse monoclonal anti-beta-catenin (1:100, BD Transduction Lab). Cell proliferation analysis was performed on paraffin-embedded sections using the PCNA Staining Kit (Zymed Laboratories) following the manufacturer's protocol. Cell proliferation was also evaluated by BrdU pulse labeling. BrdU was injected intraperitoneally into pregnant mice 4 h before death and detected using the Zymed BrdU staining kit (Zymed Laboratories) following the manufacturer's protocol.

### *In Vivo *Methylation Assay

Subconfluent 293T cells seeded on 100 mm diameter TC dishes were transfected with indicated vectors. Protein synthesis inhibition and *in vivo *methylation were carried out as described[13]. Cells were washed with ice-cold phosphate-buffered saline and harvested and lysed in 1 ml of ice-cold lysis buffer (50 mM Tris, pH 7.5, 150 mM NaCl, 0.1% Nonidet P-40, 1 mM phenylmethylsulfonyl fluoride, 5 mM NaF, 1 mM Na_3_VO_4_) supplemented with protease inhibitors (Sigma). Cell lysates were quantified using the standard Bedford method([29] and 1 mg incubated with 8 mg of A-Sepharose (Amersham Biosciences). Beads were then washed three times in ice-cold lysis buffer, and bound proteins were solubilized by addition of SDS sample buffer. Proteins were separated by SDS-PAGE and transferred to nitrocellulose membranes. Western blot analyses were performed by standard procedures with an anti-Flag antibody body (M2; Sigma) and enhanced chemiluminescence visualization. Radioactivity (methyl-^3^H) was visualized by fluorography; membranes were soaked in NAMP100 Amplify (Amersham Biosciences), air dried, and exposed to films at -80°C for 1 month.

### *In Vitro *Methylation

*In vitro *methyltransferase assays and fluorography of methylation reactions were carried out as described[25]. Experiments were performed as described using the Histone Methyltransferase Assay Protocol for active CARM1/PRMT4 protein (Upstate). Recombinant wild type and mutant Sox9 proteins (1 μg) were added to HMT reaction buffer containing 1 mM PMSF, 50 mM Tris-HCl (pH 9.0), 0.5 mM DTT and 1 mM [^3^H]AdoMet (0.5 Ci/mmol), and incubated with 700 ng of recombinant CARM1 protein (Upstate) at 30°C for 30 min in a total volume of 50 μl.

### Purification of Recombinant Proteins

Sox9 was synthesized using the BaculoDirect system, according to the manufacturer's protocol (Invitrogen). Recombinant Flag-tagged Sox9 was immunoprecipitated by anti-Flag M2 affinity gel with immunoprecipitation buffer 0 (50 mM Tris-HCl, pH 7.5, 100 mM NaCl, 0.05% Nonidet P-40, 1 mM EDTA, 2.5 mM EGTA, 10% glycerol, protease inhibitors) after indicated treatments. Purified proteins were assessed by silver staining (Bio-Rad) and Western blotting analyses. Western blotting were performed using anti-Flag M2 (Sigma) or anti-Sox9 (Chemicon) antibodies as described previously[16].

### *In situ *Hybridization

E14.5 to E18.5 embryos and neonatal P1 mice were fixed in 10% formalin for 24 h or more (depending on size). After dehydration with increasing concentrations of ethanol, embryos were embedded in paraffin, sectioned at 3 to 4 -μm, and placed on silane-treated slides. Probes were labeled with ^35^S-rUTP to a specific activity of 105 cpm/μl. ISH were performed with the following modifications. Sections were post-fixed in 4% formaldehyde, treated with 10 μg/ml proteinase K for 7.5 min, and treated with 0.1 M triethanolamine containing 0.25% acetic anhydride for 10 min. Slides were heated at 75°C and cooled on ice before hybridization. The hybridization mix containing 50% formamide, 0.3 M NaCl, 1× Denhardt's solution, 5 mM EDTA, 10 mM Tris, 10% dextran, 10 μM DTT, 10 mM NaHPO_4_, 500 μg/ml tRNA, ~200 g/ml calf thymus DNA, and the denatured probe was added to each slide and incubated at 65°C overnight. Sections were treated with RNAse at 37°C using 20 μg/ml RNAse A and 1 μg/ml RNAse T1. After washing, slides were dipped in a 50% mixture of NTB-2 (Eastman Kodak) and 0.6 M NH4Ac, further exposed at 4°C for 3-4 day for autoradiography, and subsequently developed. Sections were counter-stained with hematoxylin. The *Cyclin D1 *probe was amplified by PCR and inserted into the PCRII topo-vector (Invitrogen) (data not shown).

## Authors' contributions

TI carried out all the molecular genetic studies and drafted the manuscript. NY, JL and MTB created null and Tg mice and participated in research design. TF, SY, KY, NT, MH, MT, TO and ML contributed to histological studies of bone phenotype. HA conceived of the study, and participated in its design and coordination and helped to draft the manuscript. All authors read and approved the final manuscript.

## Supplementary Material

Additional file 1 **Expression of chondrocyte and osteoblast markers and signaling molecules in CARM1-null mutant embryos**. Sections of humerus of E16.5 heterozygous and mutant embryos are hybridized with *Col2a1, Col10a1, BSP, Op, Runx2 *and *Oc *probes. Plasmids for BSP, Op, Runx2 and Oc mRNA probes were kindly supplied by Dr. K. Nakashima. Expression of chondrocyte markers in E14.5 wt and Tg embryos. Sections of humerus of wt and Tg E14.5 embryos are hybridized with *Col2a1 *and *Col10a1 *probes. Click here for file

Additional file 2 **Generation and characterization of v5-CARM1 transgenic mice**. A) Human CARM1 cDNA under control of the cytomegalovirus immediate early enhancer-chicken beta-actin hybrid promoter (pCAGGS expression vector) was used to generate a transgenic line by male pronuclei microinjection. The pCAGGS expression plasmid displays high activity and ubiquitous expression in transgenic mouse experiments. V5 and 6 × His tags were introduced at the CARM1 C-terminus. B) V5-CARM1 is expressed at levels equivalent to endogenous CARM1; thus CARM1 levels are doubled in this model. CARM1 was immunoprecipitated from brain extracts and Western analysis performed using an anti-V5 antibody to detect V5-CARM1 in the transgenic line. The blot was stripped and re-probed with an anti-CARM1 antibody. The asterisk marks the IgG heavy chain. Click here for file
