# Supplementary figures and images for: Arginine methyltransferase CARM1/PRMT4 regulates endochondral ossification
Source: BMC Dev Biol. 2009 Sep 2;9:47. doi: 10.1186/1471-213X-9-47 (PMC2754437; doi:10.1186/1471-213X-9-47)

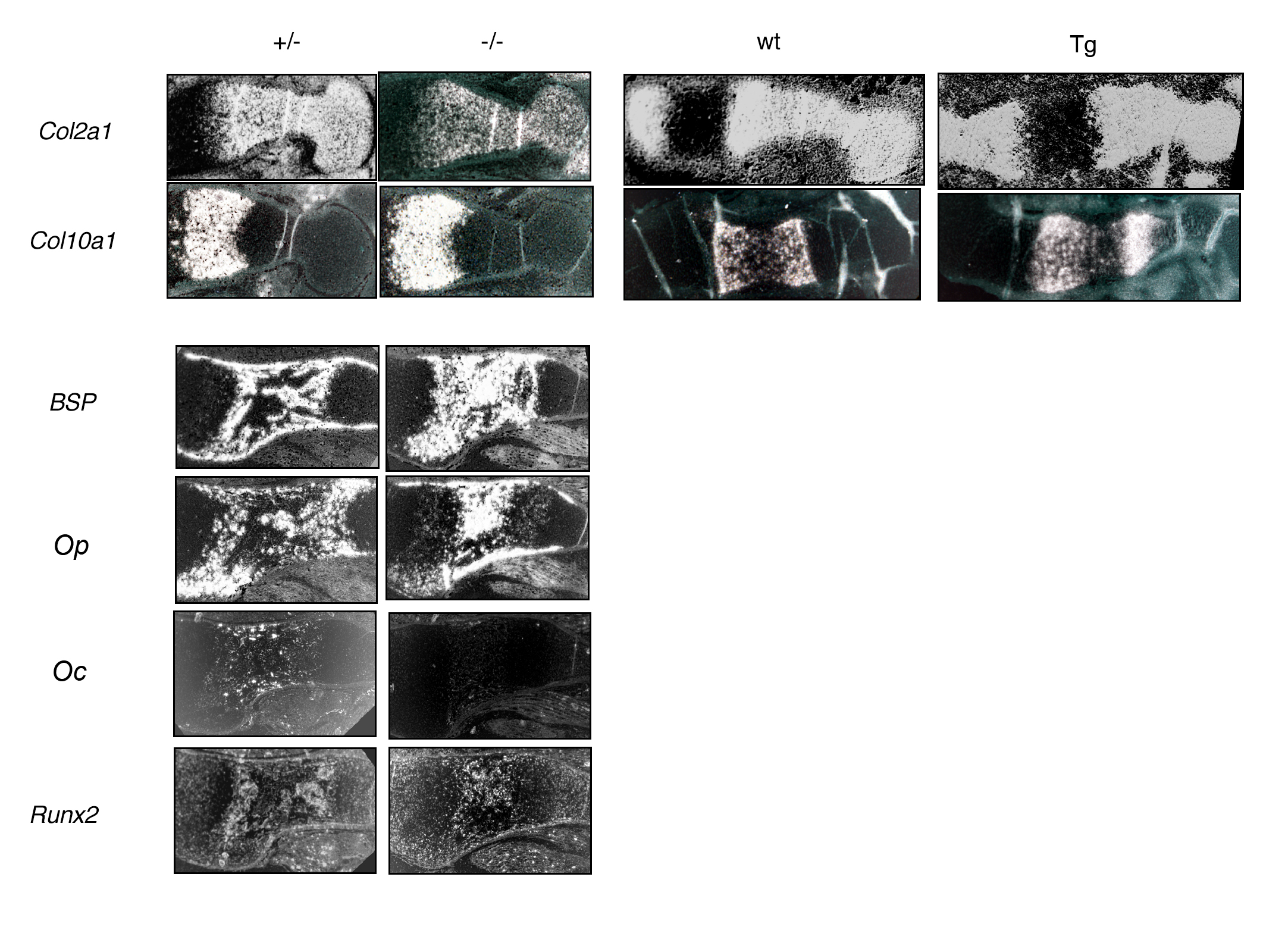

Supplement: Additional file 1 — Expression of chondrocyte and osteoblast markers and signaling molecules in CARM1-null mutant embryos. Sections of humerus of E16.5 heterozygous and mutant embryos are hybridized with Col2a1, Col10a1, BSP, Op, Runx2 and Oc probes. Plasmids for BSP, Op, Runx2 and Oc mRNA probes were kindly supplied by Dr. K. Nakashima. Expression of chondrocyte markers in E14.5 wt and Tg embryos. Sections of humerus of wt and Tg E14.5 embryos are hybridized with Col2a1 and Col10a1 probes. [file 1471-213X-9-47-S1.jpeg]

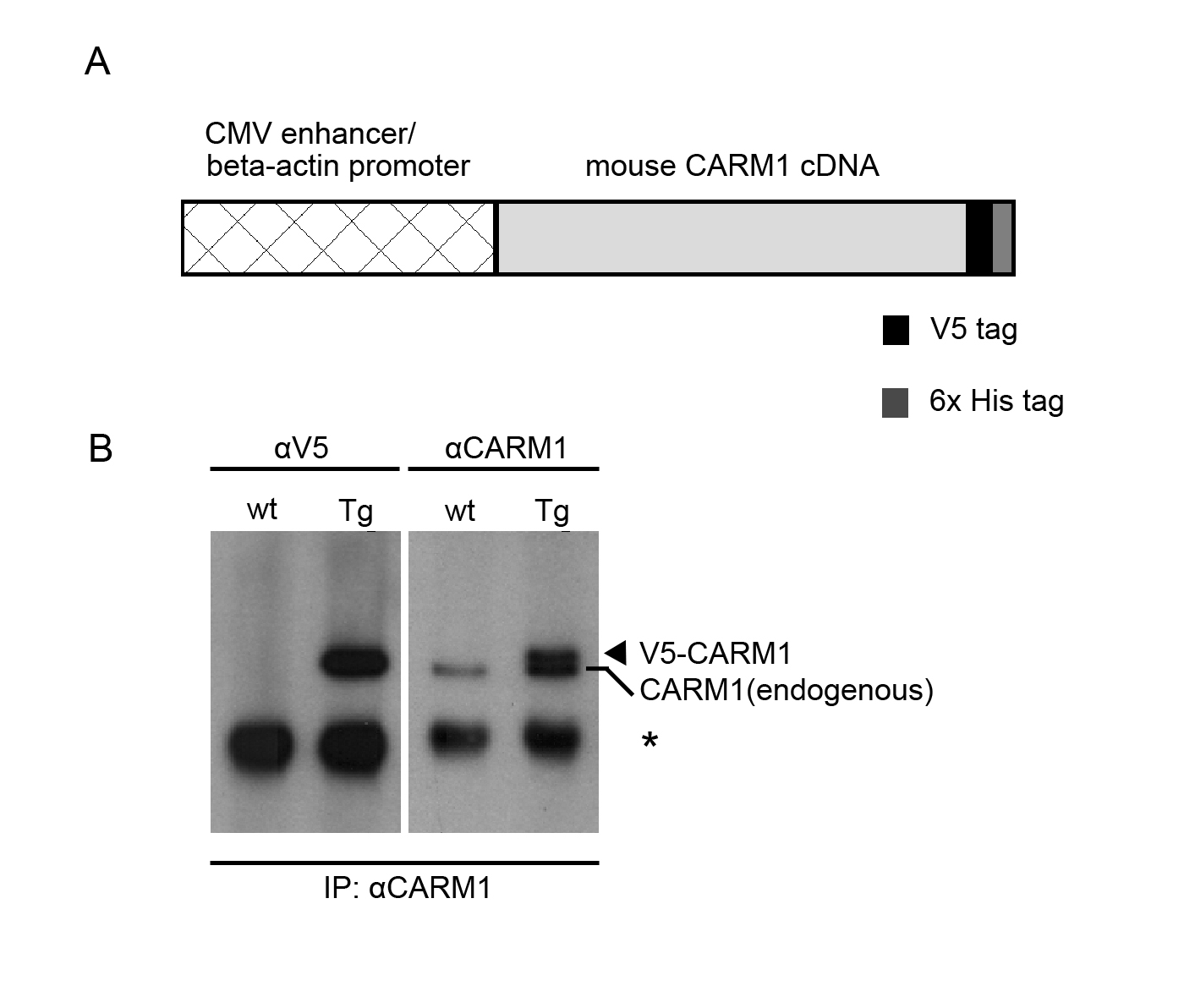

Supplement: Additional file 2 — Generation and characterization of v5-CARM1 transgenic mice. A) Human CARM1 cDNA under control of the cytomegalovirus immediate early enhancer-chicken beta-actin hybrid promoter (pCAGGS expression vector) was used to generate a transgenic line by male pronuclei microinjection. The pCAGGS expression plasmid displays high activity and ubiquitous expression in transgenic mouse experiments. V5 and 6 × His tags were introduced at the CARM1 C-terminus. B) V5-CARM1 is expressed at levels equivalent to endogenous CARM1; thus CARM1 levels are doubled in this model. CARM1 was immunoprecipitated from brain extracts and Western analysis performed using an anti-V5 antibody to detect V5-CARM1 in the transgenic line. The blot was stripped and re-probed with an anti-CARM1 antibody. The asterisk marks the IgG heavy chain. [file 1471-213X-9-47-S2.jpeg]
